# Antimicrobial Potential and Phytochemical Screening of *Clathria* sp. 1 and *Tedania* (*Tedania*) *stylonychaeta* Sponge Crude Extracts Obtained from the South East Coast of South Africa

**DOI:** 10.1155/2021/6697944

**Published:** 2021-02-26

**Authors:** Wasswa Cuthbert Kibungu, Anna-Maria Clarke, Justine Fri, Henry Akum Njom

**Affiliations:** ^1^Microbial Pathogenicity and Molecular Epidemiology Research Group (MPMERG), Department of Biochemistry and Microbiology, Department of Biochemistry & Microbiology, University of Fort Hare, Private Bag X1314, Alice 5700, South Africa; ^2^Department of Microbiology, Faculty of Natural and Agriculture, Science, North West University, Private Bag X2046, Mmabatho 2375, South Africa; ^3^Agricultural Research Council, Private Bag X1251, Potchefstroom 2531, South Africa

## Abstract

Scientists have continuously searched for novel bioactive compounds to overcome the inherent problems associated with drug resistance, the evolution of unknown diseases, and the toxicity of currently used compounds. The ocean has been considered a rich source of compounds that possess unique chemical structures and novel biological capabilities. Biologically active molecules isolated from marine flora and fauna have shown significant advancement over the past century in the pharmaceutical industry. Marine natural products (MNPs) have been used as nanomedicine, cosmetics, wound healing, antimicrobial agents, anticancer agents, and anti-inflammatory agents. The physicochemical parameters of the collection site were also recorded. This study's marine sponge species were collected from Phillip's Reef, South Africa, at 12 m during the spring season. Ethyl acetate (EA) and dichloromethane : methanol (DCM : ME, 1 : 1) were used as extraction solvents. Crude extracts of the marine sponges were tested against MRSA, *P. aeruginosa*, *C. difficile*, *A. fumigatus*, and *C. albicans*. Phytochemical screening was conducted to identify seven critical phytochemical groups. A pH reading of 8.01 and a temperature of 15.45°C were recorded at the sampling site. *Clathria* sp. 1 and *Tedania* (*Tedania*) *stylonychaeta* EA crude extracts showed bioactivity against all five test pathogens. The DCM : ME crude extract of *Clathria* sp. 1 was the only bioactive crude extract from DCM : ME extracts. This crude extract was only bioactive against *C. albicans* as no activity was observed against the other four pathogens. EA crude extracts of *Clathria* sp. 1 yielded more significant inhibition zones against both fungal pathogens. These EA crude extracts performed better than fluconazole as inhibition zones of 35 ± 0 mm at 24 mg/ml, 31 ± 0 mm at 19 mg/ml, 31 ± 0 mm at 14.4 mg/ml, 30 ± 0 mm at 9.6 mg/ml, and 25 ± 0 mm at 7.2 mg/ml were recorded. *Clathria* sp. 1 crude extracts exhibited higher inhibition zones compared to *Tedania* (*Tedania*) *stylonychaeta*. The antibiotic imipenem (26 ± 0.7 mm at 10 *μ*g) and ciprofloxacin (30 ± 0.3 mm at 5 *μ*g) exhibited higher zones of inhibition than EA crude extracts of *Tedania* (*Tedania*) *stylonychaeta* at all test concentrations. In this study, *Clathria* sp. 1 was observed to have broad-spectrum bioactivity as EA crude extracts were bioactive against MRSA, *P. aeruginosa*, *C. difficile*, *A. fumigatus*, and *C. albicans*. In addition to this, the EA crude extract of *Clathria* sp. 1 was bacteriostatic (9.6 mg/ml). *Clathria* sp. 1 DCM : ME crude extract only tested positive for the presence of terpenoids. In contrast, EA crude extracts did not test positive for the existence of any of the seven phytochemicals. Our study has revealed that *Tedania* (*Tedania*) *stylonychaeta* and *Clathria* sp. 1 sponge species collected from Phillip's Reef in South Africa can produce bioactive compounds useful against bacterial and fungal species.

## 1. Introduction

The ocean is a rich source of compounds possessing novel structures and biological activities. Biologically active molecules isolated from marine flora and fauna have applications in pharmaceuticals, cosmetics, nutritional supplements, enzymes, molecular probes, fine chemicals, and agrochemicals. Important secondary metabolites, including antibiotics, herbicides, and growth-promoting substances, are produced by several members of marine microorganisms [1]. Scientists have continuously searched for novel bioactive compounds to overcome drug resistance's inherent problems, the toxicity of currently used compounds, and the increased incidence of severe diseases, which have been reported to cause cancer, antimicrobial resistance, and neurodegenerative pathologies which have led to the need for the discovery and development of novel medicines.

Sponges are spineless animals belonging to the phylum Porifera and serve as the most primitive multicellular animals. Marine sponges are soft-bodied, sessile, and filter feeders by assembling small particulate matter from seawater rising through their bodies [2]. Marine organisms represent approximately 80% of the world's biota [3] and are a source of unique natural products ranging from food, fragrances, pigments, and insecticides. Approximately 10,000 pharmacologically bioactive compounds have been extracted from marine invertebrates. Such invertebrates are tunicates, sponges, soft corals, sea hares, nudibranchs, bryozoans, and sea slugs [4]. As of 2003, amongst marine organisms, the largest number of secondary metabolites isolated since 1965 have come from sponges [5], and they have been the primary source of biologically active molecules.

The first marine biodiscovery program was centred at a South African university. This program, a scuba collection of approximately a dozen marine sponges, was initiated by a Rhodes University ichthyologist team from a subtidal reef in the Tsitsikamma National Park, situated on the Southern Cape coast of South Africa during the spring of 1990 [6]. It was observed that sponge metabolites' primary biological activities were to kill or prevent the growth of microbial pathogens [7]. The secondary metabolites derived from these marine invertebrates have shown antibiotic, antiparasitic, antiviral, and anticancer activities [8]. About 5000 compounds to date were isolated from sponges worldwide, accounting for about 30% of all compounds obtained from the marine environment thus far. Approximately more than two hundred newly discovered bioactive products derived from sponges are reported yearly since the last decade [9]. It is hypothesized that sponges have used different metabolic pathways to produce diverse and unique bioactive compounds to support the marine sponges' survival. These chemicals include defence agents against microbial infection and agents used in the competition for space in the crowded marine reef environment [10]. Several medications derived from sponges are available in the market, and others are in clinical trials (Eribulin Mesylate, Cytarabine, and Vidarabine) [11]. Generally, marine invertebrates have been mostly researched for neurophysiological and anticancer properties rather than antimicrobial potential. Therefore, there is a lack of data on the antibacterial and antifungal activity of marine invertebrates' compounds. Most of the research done in South Africa focuses on the isolation of marine-associated microbiota for antimicrobial compound screening. Given South Africa's rich biodiversity of marine invertebrates across its far-stretching coastline, this was a perfect opportunity for marine natural products to be explored from two marine sponge species indigenous to the Southern East Coastline of South Africa.

## 2. Materials and Methods

### 2.1. Sample Collection and Taxonomical Identification

**Figure 1:**
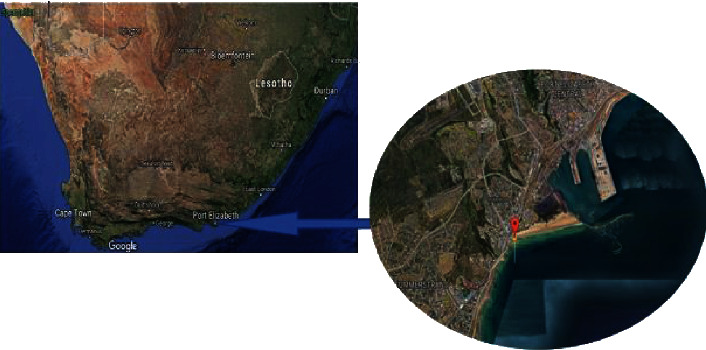
The geographical location of Phillip's Reef in Algoa Bay, Port Elizabeth, South Africa.

Samples were collected from Phillip's Reef in Algoa bay with coordinates 33°58′37.2^″^S 25°40′22.8^″^E, Port Elizabeth, Eastern Cape Province of South Africa (Figure 1).

*Clathria* sp. 1 and *Tedania* (*Tedania*) *stylonychaeta* species were collected by Scuba divers at depths of 12 m once-off during spring (12 September 2017). The site's physicochemical parameters during each collection were recorded using a Conductivity Temperature Depth (CTD) device (Seabird 19plus V2). Each sample was transferred into a sterile zip lock bag containing seawater and was maintained at 4°C. Samples were transported to the Microbial Pathogenicity and Molecular Epidemiology Research Group (MPMERG) Laboratory at the University of Fort Hare for further analysis. Samples were stored at -20°C before the extraction of bioactive compounds. A portion of each sponge specimen was sent to the Department of Environmental Affairs Oceans and Coastal Research Centre, Cape Town, and South African Environmental Observation Network, Port Elizabeth, South Africa (taxonomical identification). Identification was made using morphological approaches. Briefly, a section of each sponge specimen was cut and placed in household bleach to digest the sponge material. The cut sections and spicule preparations were mounted on microscope slides and allowed to dry. Permanent mounts were made using Entellan or Canada balsam. Sponge morphology, arrangement, and spicule morphology were used for identification.

### 2.2. Bioactive Compound Extraction

Ethyl acetate (EA) and dichloromethane : methanol (DCM : ME, 1 : 1) were used for the isolation of bioactive metabolites as previously described [12, 13]. For each sponge, 10 g of fresh or thawed sample was minced using a blender and added to 150 ml of each solvent (EA, DCM : ME). The tissue and solvent mixtures of EA and DCM : ME were agitated for 72 and 48 hours, respectively, followed by filtration through a Whatman No. 2 filter paper. The filtrates were concentrated using a rotary vacuum evaporator (Lasec Steroglass, Strike 202) at 40°C at 130 rpm. The resulting crude extract residues, approximately 1 ml per 50 ml of evaporated solution, were stored at 4°C for further analysis.

### 2.3. Test Organisms/Growth

Commercial strains of bacteria, methicillin-resistant *Staphylococcus aureus* ATCC 49476 and *Pseudomonas aeruginosa* ATCC 10145, were resuscitated on Brain Heart Infusion (BHI) agar (Oxoid, UK) at 37°C for 24 hours [14]. *Clostridium difficile* ATCC 9689 was resuscitated with BHI supplemented with 5 mg ml^−1^ of yeast extract and incubated at 37°C for 48 hours microaerophilically [15]. Fungal species of *Candida albicans* ATCC 10231 and *Aspergillus fumigatus* ATCC 204305 were cultured on Potato Dextrose Agar (PDA) and incubated at 30°C for three to five days [16].

### 2.4. Antimicrobial Assays

The antibacterial activity of the crude extracts obtained was evaluated using the standard agar well diffusion assay on Muller Hinton Agar (MHA) with slight modifications [17]. Inoculums of the test bacteria, methicillin-resistant *S. aureus* ATCC 49476, *C. difficile* ATCC 9689, and *P aeruginosa* ATCC 10145, were prepared by transferring colonies from an overnight culture into 0.9% normal saline and turbidity adjusted to 0.5 McFarland standards (the equivalent of one to two ×108 cfu ml^−1^). Two hundred and fifty microliters of each adjusted bacteria suspension was introduced into 500 ml of MHA (Oxoid, UK), which was cooled to about 40°C post sterilization. The mixture was poured into Petri dishes and allowed to solidify. Wells of six mm diameter were punched into the MHA plates containing test bacteria using a sterile cork borer. Crude extract concentrations of 80% (*v* : *v*) were prepared. The experiments were done in triplicates for all crude extracts obtained using ethyl acetate and dichloromethane : methanol (1 : 1) (*v* : *v*). Twenty microliters of each of the extracts was dispensed into different wells, and a negative control well was filled with the corresponding solvents. Antibiotic disks were also included as positive controls in the assays. Vancomycin (VN, 30 *μ*g), clindamycin (CD, 10 *μ*g), trimethoprim (TM, 5 *μ*g), rifampicin (RP, 5 *μ*g), and amoxicillin (AMX, 25 *μ*g) were tested against MRSA. Imipenem (IMP, 10 *μ*g), ciprofloxacin (CIP, 5 *μ*g), and meropenem (MEM, 10 *μ*g) was tested against *P. aeruginosa*. Metronidazole (MNZ, 5 *μ*g), tetracycline (TE, 30 *μ*g), and clarithromycin (CLR, 15 *μ*g) were tested against *C. difficile*. *S. aureus* and *P. aeruginosa* inoculated plates were incubated at 37°C for 24 hours. Those of *C. difficile* were incubated at 37°C for 48 hours under microaerophilic conditions. The resulting diameters of inhibition zones were recorded in millimetres.

The antifungal property of different extracts against the test fungi, *C. albicans* (ATCC 10231) and *A. fumigatus* (ATCC 204305), was determined as previously reported by Selvin and Lipton [17]. However, the media used was PDA. Negative controls were wells filled with corresponding solvents while the antifungal agents, fluconazole (FLU, 15 *μ*g), itraconazole (ICZ, 10 *μ*g), and voriconazole (VCZ, 5 *μ*g), were included as positive controls. Incubation was done at 37°C for three to five days [18]. All assays were done in triplicate with a total dry weight of 24.5 mg being added into 1 ml of the parent extraction solvent to create a 24 mg/ml stock solution.

### 2.5. Phytochemical Screening of Bioactive Crude Extracts

Crude extracts that exhibited antimicrobial activity against the bacteria and fungi were tested for phytochemical compounds such as flavonoids, tannins, terpenoids, phenolic compounds, saponins, and alkaloids.

#### 2.5.1. Detection of Flavonoids

Flavonoids were detected using the Juglone test method described by Sofowora [19]. Diluted ammonia solution was added to the crude filtrate, followed by adding 1 ml of concentrated sulphuric acid and 2 ml of potassium hydroxide solution, and allowed to mix. 1 ml of aqueous filtrate of the sample was added before observing visible color changes.

#### 2.5.2. Detection of Tannins

Detection of tannins was evaluated as reported by Iyengar [20]. A few drops of 0.1% ferric chloride were added to 0.5 ml of crude filtrate and observed for brownish green or blue-black coloration, which indicated the presence of tannins.

#### 2.5.3. Detection of Terpenoids

The Salkowski test was used to detect terpenoids, as described by Kiran et al. [21]. To a volume of 5 ml of crude filtrate, 2 ml of chloroform was added. Then, 3 ml of concentrated sulphuric acid was added. A reddish-brown coloration of the interface indicated the presence of terpenoids.

#### 2.5.4. Detection of Phenolic Compounds

The method reported by Mace [22] was followed. A volume of 2 ml of the crude filtrate and alcohol was mixed with a few drops of neutral ferric chloride (pH 7) solution. A dark green color indicated the presence of a phenolic group.

#### 2.5.5. Detection of Saponins

Saponins were detected using the method described by Venkatesh et al. [23]. In this test, 0.5 ml of latex was dissolved in 5 ml of distilled water in a test tube. The solution was shaken vigorously and observed for a stable, persistent froth with a honeycomb structure.

#### 2.5.6. Detection of Anthraquinones

The crude filtrate (2.5 ml) was shaken with 5 ml of benzene and 2.5 mL of 10% ammonia solution. A pink, red, or violet color indicated the presence of anthraquinones, as reported by Mujeeb et al. [24].

#### 2.5.7. Detection of Alkaloids

Samples containing alkaloids were identified using Evans's method [25]. A volume of 2 ml of 1 M hydrochloric acid was added to 1 ml of the crude filtrate. A few drops of Mayer's reagent (mercuric chloride 1.36 g, potassium iodide 5.0 g dissolved separately, and the volume made up to 100 ml with distilled water) were added to the test tube. A white or creamy precipitate indicated a positive result for alkaloids.

### 2.6. Statistical Analysis

Means of zones of inhibition obtained from the bioactivity assays were derived and the standard deviations calculated using Microsoft Excel. A two-way Analysis of Variance (ANOVA) was carried out to determine if there were any statistical differences between the mean zones of inhibition of the ethyl acetate and dichloromethane : methanol crude extracts of the sponges collected during the spring against the pathogens of interest. The GraphPad Prism version 7.04 (Prime 7 for Windows) Microsoft Software was used for this analysis. The significance value was set at *p* < 0.05.

## 3. Results and Discussion

### 3.1. Results

#### 3.1.1. Environmental Conditions of Phillip's Reef, South Africa

**Figure 2:**
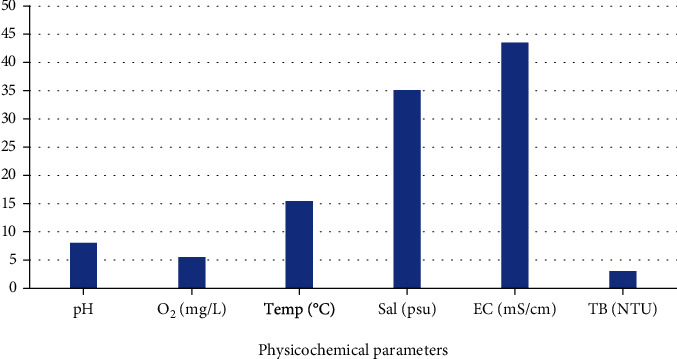
Physicochemical conditions of Phillip's Reef, South Africa.

Sponge species were collected during spring; the physicochemical conditions of the sample collection site were evaluated. A pH reading of 8.01, total dissolved oxygen of 5.52 O_2_ mg·ml^−1^, temperature of 15.45°C, electroconductivity of 43.5 mS·cm^−1^, and turbidity of 3.04 NTU were recorded.  Figure 2 illustrates Phillip's Reef's physicochemical conditions at a depth of 12 m.

#### 3.1.2. Taxonomic Identification and Seasonal Distribution of Marine Invertebrates

**Figure 3:**
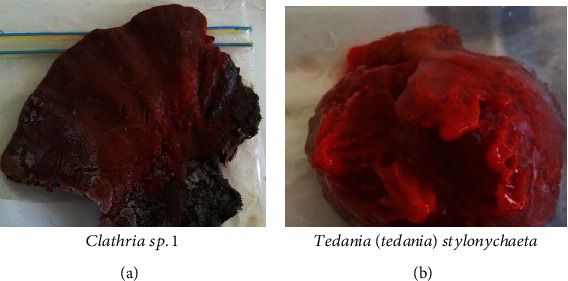
Images of some marine invertebrate species harvested from Phillip's Reef in Algoa Bay, Port Elizabeth.

Two marine invertebrate species were collected at Phillip's Reef, Port Elizabeth, during spring (Figures 3(a) and 3(b)).

#### 3.1.3. Antimicrobial Activity of Crude Extracts of *Clathria* sp. 1 and *Tedania* (*Tedania*) *stylonychaeta*, Marine Invertebrate Species

*Clathria* sp. 1 and *Tedania* (*Tedania*) *stylonychaeta* EA crude extracts showed bioactivity against all five test pathogens. The DCM : ME crude extract of *Clathria* sp. 1 was the only bioactive crude extract from DCM : ME extracts. This crude extract was only bioactive against *C. albicans* as no activity was observed against the other four pathogens. The EA crude extract of *Clathria* sp. 1 (35 ± 0 mm at 24 mg/ml concentration) still performed better than the DCM : ME (19 ± 0 mm at 24 mg/ml concentration) crude extract when tested on *C. albicans*. Against MRSA, EA crude extract of *Clathria* sp. 1 (16.5 ± 0.7 mm) showed better bioactivity at 7.2 mg/ml than EA crude extract of *Tedania* (*Tedania*) *stylonychaeta* (11 ± 0 mm). Both crude extracts showed the same MIC against MRSA but different MBCs. *Clathria* sp. 1 EA crude extracts were not bacteriostatic against MRSA, although high inhibition zones were observed. At a concentration of 24 mg/ml, both EA crude extracts of *Clathria* sp. 1 (22.5 ± 0.7 mm) *Tedania* (*Tedania*) *stylonychaeta* (20 ± 0 mm) exhibited higher zones of inhibition. These zones were more elevated than amoxicillin (11 ± 1.2 mm at 25 *μ*g) and vancomycin (18 ± 1.2 mm at 30 *μ*g).

*Tedania* (*Tedania*) *stylonychaeta* EA crude extracts were only bioactive against *P. aeruginosa* at 24 mg/ml, 19 mg/ml, and 14.4 mg/ml as inhibition zones of 17 ± 1.4 mm, 15.5 ± 0.7 mm, and 12 ± 2.8 mm were recorded, respectively. In contrast to this, EA crude extracts of *Clathria* sp. 1 showed activity at all test concentrations. Higher zones of inhibition were observed with *Clathria* sp. 1 (30 ± 0 mm at 24 mg/ml, 27 ± 0 mm at 19 mg/ml, 26 ± 1.4 mm at 14.4 mg/ml, 26 ± 1.4 mm at 9.6 mg/ml, and 25.5 ± 2.1 mm at 7.2 mg/ml) in comparison to *Tedania* (*Tedania*) *stylonychaeta* (17 ± 1.4 mm at 24 mg/ml, 15.5 ± 0.7 mm at 19 mg/ml, and 12 ± 2.8 mm at 14.4 mg/ml) as seen in Table 1. *Tedania* (*Tedania*) *stylonychaeta* was bacteriostatic against *P. aeruginosa* just as observed against MRSA at a 19 mg/ml concentration. *Clathria* sp. 1 EA crude extract did not show any bacteriostatic activity, although a MIC at 7.2 mg/ml was observed. Ciprofloxacin (5 *μ*g) was the best performing control antibiotic against *P. aeruginosa* as an inhibition zone of 30 ± 0.3 mm was recorded. A similar inhibition zone of 30 ± 0 mm at 24 mg/ml was obtained from *Clathria* sp. 1. Imipenem (26 ± 0.7 mm at 10 *μ*g) and ciprofloxacin (30 ± 0.3 mm at 5 *μ*g) exhibited higher zones of inhibition than EA crude extracts of *Tedania* (*Tedania*) *stylonychaeta* at all test concentrations.

A different bioactivity profile was observed with *Clathria* sp. 1 EA crude extract against *C. difficile*. At 24 mg/ml and 7.2 mg/ml, no bioactivity was observed against *C. difficile*. Instead, at 19 mg/ml (25 ± 0 mm), 14.4 mg/ml (23 ± 0 mm) and 9.6 mg/ml (16 ± 0 mm) bioactivity were recorded. Considering this, these zones of inhibition were still higher than those obtained from *Tedania* (*Tedania*) *stylonychaeta* at concentrations 24 mg/ml to 7.2 mg/ml (15 ± 0 mm, 15 ± 0 mm, 13 ± 0 mm, 12 ± 0 mm, and 10 ± 0 mm). No bactericidal activity was observed from EA crude extract of *Clathria* sp. 1 even though the MIC of *Clathria* sp. 1 (9.6 mg/ml) was the same as EA crude extract of *Tedania* (*Tedania*) *stylonychaeta*. Clarithromycin (40 ± 0.1 mm at 15 *μ*g) showed greater bioactivity than *Clathria* sp. 1 (25 ± 0 mm at 19 mg/ml) and *Tedania* (*Tedania*) *stylonychaeta* (15 ± 0 mm at 19 mg/ml) EA crude extracts. Only EA crude extracts of *Clathria* sp. 1 exhibited bioactivity against *A. fumigatus*. Inhibition zones of 22 ± 0 mm at 24 mg/ml, 18 ± 1.4 mm at 19 mg/ml, 17.5 ± 0.7 mm at 14.4 mg/ml, 13.5 ± 0.7 mm at 9.6 mg/ml, and 13 ± 0 mm at 7.2 mg/ml were recorded. The MIC for this crude extract was obtained at 7.2 mg/ml. EA and DCM : ME crude extracts of *Clathria* sp. 1 were bioactive against *C. albicans*. The DCM : ME crude extract was only bioactive at 24 mg/ml as an inhibition zone of 19 ± 0 mm was obtained. On the other hand, the EA crude extracts of *Clathria* sp. 1 exhibited almost double the DCM inhibition zone : ME crude extract as an inhibition zone of 35 ± 0 mm was obtained at 24 mg/ml. From all crude extracts tested against *C. albicans*, EA crude extract of *Clathria* sp. 1 was bacteriostatic (9.6 mg/ml).

**Figure 4:**
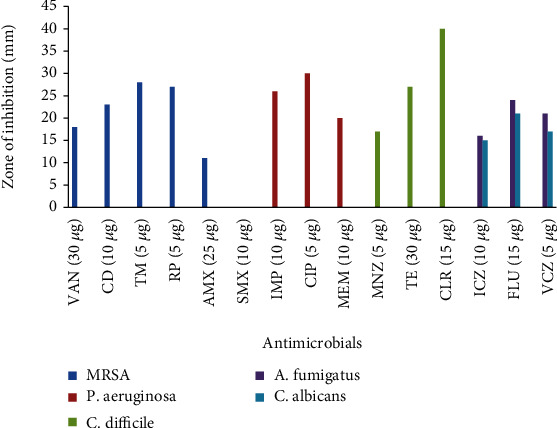
Control antimicrobials tested against all 5 test pathogens.

The EA crude extracts of *Clathria* sp. 1 and *Tedania* (*Tedania*) *stylonychaeta* recorded the same MIC at 7.2 mg/ml. EA crude extracts of *Tedania* (*Tedania*) *stylonychaeta* recorded inhibition zones of 20 ± 0 mm at 24 mg/ml, 16 ± 0 mm at 19 mg/ml, 16 ± 0 mm at 14.4 mg/ml, 16 ± 0 mm at 9.6 mg/ml, and 13 ± 0 mm at 7.2 mg/ml. All these inhibition zones were less than those recorded by EA crude extract of *Clathria* sp. 1 at all test concentrations. Fluconazole (15 *μ*g) recorded the highest zone of inhibition against both *C. albicans* (21 ± 0.2 mm) and *A. fumigatus* (24 ± 2.1 mm). The inhibition zone obtained from fluconazole was greater than that recorded from EA crude extracts of *Tedania* (*Tedania*) *stylonychaeta* at all test concentrations. However, EA crude extracts of *Clathria* sp. 1 yielded greater inhibition zones against both fungal pathogens than fluconazole. Inhibition zones of 35 ± 0 mm at 24 mg/ml, 31 ± 0 mm at 19 mg/ml, 31 ± 0 mm at 14.4 mg/ml, 30 ± 0 mm at 9.6 mg/ml, and 25 ± 0 mm at 7.2 mg/ml were recorded, as seen in Tables 2, 3 and 1 and Figure 4.

#### 3.1.4. Phytochemical Screening of Collected Marine Sponge Species

Seven phytochemicals were screened for this study (tannins, phenolics, terpenoids, anthraquinones, saponins, flavonoids, and alkaloids) as seen in Table 4, from the crude extracts obtained from 2 marine species exhibiting bioactivity. *Clathria* sp. 1 DCM : ME crude extract only tested positive for the presence of terpenoids. EA crude extracts did not test positive for the existence of any of the seven phytochemicals. A similar observation was observed with the EA crude extracts of *Tedania* (*Tedania*) *stylonychaeta* as it only tested positive for flavonoids.

### 3.2. Discussion

The discovery of useful antimicrobial compounds is gaining ground. Antimicrobial resistance is an existing problem and necessitates alternative therapeutic agents for disease management [26]. Substantial work has been done on terrestrial sources of bioactive compounds, such as those derived from plants, which has led to many novel medicines [27]. Since the ocean is home to the largest percentage of living organisms, it serves as a prolific source of novel antimicrobials. Marine invertebrates have been proven in some parts of the world as good sources of antifungal, antibacterial, larvicidal, anticancer, and anti-inflammatory compounds [28]. Phillip's Reef's environmental conditions recorded a pH reading of 8.01 and a temperature of 15.45°C, as seen in Figure 2. Our study observed that these ecological conditions were suitable for sponge bioactive metabolite production as bioactivity against MRSA, *P. aeruginosa*, *C. difficile*, *A. fumigatus*, and *C. albicans* was observed. Putra et al. [29] reported that specific marine species' bioactivity potential could be induced or suppressed by environmental stress factors such as temperature, pH, dissolved nutrients, and epibiont (pathogenic) colonization fouling. There is a lack of information present in the literature regarding the effect of environmental conditions on marine sponges' bioactivity potential.

In this study, some crude extracts of the marine invertebrates showed varying bioactivity. The EA acetate crude extracts of both *Tedania* (*Tedania*) *stylonychaeta* and *Clathria* sp. 1 showed bioactivity against most test pathogens compared to one DCM : ME crude extracts, which showed activity against *C. albicans*. Bayona et al. [30] reported that the extraction of bioactive metabolites from marine sponges depends on several factors such as pH, pressure, polarity index, and extraction technique(s) used. Therefore, it cannot be concluded that DCM : ME is a weak extraction solvent for marine bioactive metabolites. Hutagalung et al. [18] made similar observations. Furthermore, they concluded that the success of extracting sponge-based metabolites depends on the type of metabolite being extracted. The type of methods to be used to remove the desired metabolite(s), if known, is essential. Metabolites such as lipid, alkaloid, terpenoid, peptide, protein, phenols, and quinones can be extracted. Equally important is the respective extraction solvent to be used either individually or in combination with another solvent. *Clathria* sp. 1 exhibited higher inhibition zones compared to *Tedania* (*Tedania*) *stylonychaeta*. *Clathria* sp. 1 also showed broad-spectrum bioactivity as EA crude extracts were bioactive against MRSA, *P. aeruginosa*, *C. difficile*, *A. fumigatus*, and *C. albicans*. Cita et al. [31] reported that methanolic crude extracts of *Clathria* sp. collected from Pasir Putih, East Java (Indonesia), were not bioactive against *P. aeruginosa* and MRSA. In our study, contrasting results were obtained. At a concentration of 24 mg/ml, the EA crude extract of *Clathria* sp. 1 exhibited inhibition zones (22.5 ± 0.7 mm). The crude extracts yielded higher zones of inhibition than amoxicillin (11 ± 1.2 mm at 25 *μ*g) and vancomycin (18 ± 1.2 mm at 30 *μ*g) against MRSA, while high zones of inhibition observed with EA crude extracts of *Clathria* sp. 1 (30 ± 0 mm at 24 mg/ml,27 ± 0 mm at 19 mg/ml,26 ± 1.4 mm at 14.4 mg/ml,26 ± 1.4 mm at 9.6 mg/ml, and25.5 ± 2.1 mm at 7.2 mg/ml) were recorded against *P. aeruginosa*, thus illustrating the efficacy of ethyl acetate crude extracts of the South African *Clathria* sp. 1 over the Indonesian methanolic crude extracts of *Clathria* sp. Said et al. [32] reported that the species of *Tedania* did not show bioactivity against *C. albicans*, *S. aureus*, and *P. aeruginosa*. Our study recorded bioactivity against the same test pathogens. *Tedania* (*Tedania*) *stylonychaeta* EA crude extracts were bioactive against *P. aeruginosa* at 24 mg/ml, 19 mg/ml, and 14.4 mg/ml as inhibition zones of 17 ± 1.4 mm, 15.5 ± 0.7 mm, and 12 ± 2.8 mm were obtained, respectively. Against MRSA, EA crude extracts of *Tedania* (*Tedania*) *stylonychaeta* which resulted in inhibition zones of 20 ± 0 mm at 24 mg/ml were recorded. Therefore, this data further illustrates the broad-spectrum antimicrobial potential that the South African marine species of *Tedania* has over both the Indonesian sponge (*Clathria* sp.) and *Tedania* isolated in Zanzibar.

Marine sponges are known to produce diverse groups of phytochemicals, including alkaloids, diterpenoids, sesquiterpenoids, and steroids [33]. These different bioactive compounds have unique biological functions and have been identified to possess promising therapeutic applications [34]. Between 1984 and 2014, the number of new marine natural products (MNPs) reported steadily grew from 332 in 1984 to 1378 in 2014 [35, 36]. A study was done by Moore et al. [15] further proved that sponges do produce phytochemicals. In their research, alkaloids such as Manadomanzamines A and B were isolated from an Indonesian sponge, *Acanthostrongylophora* sp., that showed activity against *Mycobacterium tuberculosis*. Our study evaluated the phytochemicals present in the crude extracts of marine species bioactive against MRSA, *P. aeruginosa*, *C. difficile*, *A. fumigatus*, and *C. albicans*. *Clathria* sp. 1 DCM : ME crude extract contained terpenoids, while EA crude extracts did not test positive for the presence of any of the seven phytochemicals. EA crude extracts of *Tedania* (*Tedania*) *stylonychaeta* only tested positive for flavonoids.

In contrast to our study, Weerasinghe et al. [37] reported the absence of flavonoids in their *Clathria* sp. samples and the presence of alkaloids and saponins in their ethanolic crude extracts. Several reasons could have led to different phytochemicals being extracted, such as the type of solvent used for extraction, extraction method, and the sponge species' environmental conditions under evaluation [30]. Therefore, ethanol as an extraction solvent results in the successful extraction of alkaloids and saponins in *Clathria* sp., while ethyl acetate results in flavonoids' isolation. Diversified bioactive compounds (peptides, purine derivatives, and fatty acids) have been identified from various sponges' species [38]. These have also been reported to possess significant antibacterial activity against resistant strains of *Staphylococcus aureus*, *Pseudomonas aeruginosa*, *E. coli*, *Neisseria gonorrhea*, and *Streptococcus pneumonia* [38]. Most studies have evaluated the presence of phytochemicals in plants, with less work being done regarding evaluating the potential antimicrobial properties of sponges found in oceans.

## 4. Conclusion

Our study has revealed that *Tedania* (*Tedania*) *stylonychaeta* and *Clathria* sp. 1 sponge species collected from Phillip's Reef in South Africa can produce bioactive compounds useful against Gram-positive and Gram-negative bacteria and fungi. These bioactive compounds are just as potent as reference antimicrobials. These marine species also can synthesize biologically active phytochemicals. Screening for bioactive compounds further revealed that the method used to extract bioactive compounds depends on the metabolite of interest. Ethyl acetate was identified as an excellent extraction solvent for bioactive compounds from the two species reported in this study. Although few phytochemical groups were characterized, it may be that the sponges contain bioactive peptides, sterols, or lipids. Therefore, we recommend further studies that look at the screening of these groups of compounds. Other studies that should be undertaken should look at the cytotoxicity, antioxidant, hemolysis, and antiviral properties. The structural elucidation of purified compounds is also necessary. The results obtained from this study are the first to report antimicrobial bioactivity against human pathogens using sponge matrices. They should be replicated using the same species from different areas within the South African coastline to gain insight into the species' bioactivity profile and the geographic location's role on marine sponges' bioactivity potentials. Such information is still lacking in the literature.

## Figures and Tables

**Table 1:** Minimum inhibitory, bactericidal, and fungicidal concentrations of sponge crude extracts obtained from Phillip's reef.

Microbial species	Sponge species	Ethyl acetate		Dichloromethane : methanol	
		MIC (mg/ml)	MBC/MFC (mg/ml)	MIC (mg/ml)	MBC/MFC (mg/ml)
*Staphylococcus aureus* (MRSA)	*Clathria* sp. 1	7.2	∗∗	∗	∗∗
	*Tedania* (*Tedania*) *stylonychaeta*	7.2	9.6	∗	∗∗
*Pseudomonas aeruginosa*	*Clathria* sp. 1	7.2	∗∗	∗	∗∗
	*Tedania* (*Tedania*) *stylonychaeta*	19	14.4	∗	∗∗
*Clostridium difficile*	*Clathria* sp. 1	9.6	∗∗	∗	∗∗
	*Tedania* (*Tedania*) *stylonychaeta*	7.2	9.6	∗	∗∗
*Aspergillus fumigatus*	*Clathria* sp. 1	7.2	∗∗	∗	∗∗
*Candida albicans*	*Clathria* sp. 1	7.2	9.6	∗	∗∗
	*Tedania* (*Tedania*) *stylonychaeta*	7.2	∗∗	∗	∗∗

^∗^No MIC; ^∗∗^No MBC/MFC.

**Table 2:** Antimicrobial activity of ethyl acetate derived crude extracts from marine sponges collected during spring against selected bacterial and fungal species.

Microbial species	Sponge species	Ethyl acetate				
		Zone of inhibition at various concentrations (mm)				
		24 mg/ml	19 mg/ml	14.4 mg/ml	9.6 mg/ml	7.2 mg/ml
*Staphylococcus aureus* (MRSA)	*Clathria* sp. 1	22.5 ± 0.7	22 ± 0	20.5 ± 2.1	17 ± 2.8	16.5 ± 0.7
	*Tedania* (*Tedania*) *stylonychaeta*	20 ± 0	19 ± 0	15 ± 0	11 ± 0	11 ± 0
*Pseudomonas aeruginosa*	*Clathria* sp. 1	30 ± 0	27 ± 0	26 ± 1.4	26 ± 1.4	25.5 ± 2.1
	*Tedania* (*Tedania*) *stylonychaeta*	17 ± 1.4	15.5 ± 0.7	12 ± 2.8	0	0
*Clostridium difficile*	*Clathria* sp. 1	0	25 ± 0	23 ± 0	16 ± 0	0
	*Tedania* (*Tedania*) *stylonychaeta*	15 ± 0	15 ± 0	13 ± 0	12 ± 0	10 ± 0
*Aspergillus fumigatus*	*Clathria* sp. 1	22 ± 0	18 ± 1.4	17.5 ± 0.7	13.5 ± 0.7	13 ± 0
	*Tedania* (*Tedania*) *stylonychaeta*	0	0	0	0	0
*Candida albicans*	*Clathria* sp. 1	35 ± 0	31 ± 0	31 ± 0	30 ± 0	25 ± 0
	*Tedania* (*Tedania*) *stylonychaeta*	20 ± 0	16 ± 0	16 ± 0	16 ± 0	13 ± 0

**Table 3:** Antimicrobial activity of dichloromethane: methanol derived crude extracts from marine sponges collected during spring against selected bacterial and fungal species.

Microbial species	Sponge species	Dichloromethane : methanol (1 : 1)				
		Zone of inhibition at various concentrations (mm)				
		24 mg/ml	19 mg/ml	14.4 mg/ml	9.6 mg/ml	7.2 mg/ml
*Staphylococcus aureus* (MRSA)	*Clathria* sp. 1	0	0	0	0	0
	*Tedania* (*Tedania*) *stylonychaeta*	0	0	0	0	0
*Pseudomonas aeruginosa*	*Clathria* sp. 1	0	0	0	0	0
	*Tedania* (*Tedania*) *stylonychaeta*	0	0	0	0	0
*Clostridium difficile*	*Clathria* sp. 1	0	0	0	0	0
	*Tedania* (*Tedania*) *stylonychaeta*	0	0	0	0	0
*Aspergillus fumigatus*	*Clathria* sp. 1	0	0	0	0	0
	*Tedania* (*Tedania*) *stylonychaeta*	0	0	0	0	0
*Candida albicans*	*Clathria* sp. 1	19 ± 0	0	0	0	0
	*Tedania* (*Tedania*) *stylonychaeta*	0	0	0	0	0

**Table 4:** Phytochemicals present in bioactive crude extracts of *Clathria* sp. 1 and *Tedania* (*Tedania*) *stylonychaeta*.

	Dichloromethane : methanol							Ethyl acetate						
Species	1	2	3	4	5	6	7	1	2	3	4	5	6	7
*Clathria* sp. 1	−	−	+	−	−	−	−	−	−	−	−	−	−	−
*Tedania* (*Tedania*) *stylonychaeta*	∗	∗	∗	∗	∗	∗	∗	−	−	−	−	−	+	−

Key: S(*n*) species code number: 1: tannins; 2: phenolics; 3: terpenoids; 4: anthraquinones; 5: saponins; 6: flavonoids; 7: alkaloids; +: present; −: absent; ∗: no bioactivity of crude extract.

## Data Availability

All data used in this study are present in this article.
